# Simultaneous Quantification of Spatially Discordant Alternans in Voltage and Intracellular Calcium in Langendorff-Perfused Rabbit Hearts and Inconsistencies with Models of Cardiac Action Potentials and Ca Transients

**DOI:** 10.3389/fphys.2017.00819

**Published:** 2017-10-20

**Authors:** Ilija Uzelac, Yanyan C. Ji, Daniel Hornung, Johannes Schröder-Scheteling, Stefan Luther, Richard A. Gray, Elizabeth M. Cherry, Flavio H. Fenton

**Affiliations:** ^1^School of Physics, Georgia Institute of Technology, Atlanta, GA, United States; ^2^Max Planck Institute for Dynamics and Self-Organization, Gottingen, Germany; ^3^Center for Device and Radiological Health, Food and Drug Administration, Silver Spring, MD, United States; ^4^School of Mathematical Sciences, Rochester Institute of Technology, Rochester, NY, United States

**Keywords:** discordant alternans, calcium dynamics, voltage-calcium coupling, arrhythmia, optical mapping, long QT syndrome, cardiac cell modeling

## Abstract

**Rationale:** Discordant alternans, a phenomenon in which the action potential duration (APDs) and/or intracellular calcium transient durations (CaDs) in different spatial regions of cardiac tissue are out of phase, present a dynamical instability for complex spatial dispersion that can be associated with long-QT syndrome (LQTS) and the initiation of reentrant arrhythmias. Because the use of numerical simulations to investigate arrhythmic effects, such as acquired LQTS by drugs is beginning to be studied by the FDA, it is crucial to validate mathematical models that may be used during this process.

**Objective:** In this study, we characterized with high spatio-temporal resolution the development of discordant alternans patterns in transmembrane voltage (V_m_) and intracellular calcium concentration ([Ca_i_]^+2^) as a function of pacing period in rabbit hearts. Then we compared the dynamics to that of the latest state-of-the-art model for ventricular action potentials and calcium transients to better understand the underlying mechanisms of discordant alternans and compared the experimental data to the mathematical models representing V_m_ and [Ca_i_]^+2^ dynamics.

**Methods and Results:** We performed simultaneous dual optical mapping imaging of V_m_ and [Ca_i_]^+2^ in Langendorff-perfused rabbit hearts with higher spatial resolutions compared with previous studies. The rabbit hearts developed discordant alternans through decreased pacing period protocols and we quantified the presence of multiple nodal points along the direction of wave propagation, both in APD and CaD, and compared these findings with results from theoretical models. In experiments, the nodal lines of CaD alternans have a steeper slope than those of APD alternans, but not as steep as predicted by numerical simulations in rabbit models. We further quantified several additional discrepancies between models and experiments.

**Conclusions:** Alternans in CaD have nodal lines that are about an order of magnitude steeper compared to those of APD alternans. Current action potential models lack the necessary coupling between voltage and calcium compared to experiments and fail to reproduce some key dynamics such as, voltage amplitude alternans, smooth development of calcium alternans in time, conduction velocity and the steepness of the nodal lines of APD and CaD.

## Introduction

Long-QT syndrome (LQTS), characterized by abnormal prolongation of the QT interval (Schwartz et al., 1993), is a result of delayed repolarizations in the heart and can increase the risk of life-threatening arrhythmias, with a mortality rate of 20% within the first year after first detection and up to 50% in the next 10 years for untreated patients (Schwartz, 1985). The known dangers of LQTS have resulted in guidelines by the FDA concerning the design and testing of *any* new drug and in the interpretation and analysis of these drugs in clinical trials (Food and Drug Administration, 2005). In addition, many currently available medications can be very dangerous to some patients with heart problems, as they are known to further prolong QT intervals as shown in the compendium maintained by the Sudden Arrhythmia Death Syndromes Foundation (sads.org). LQTS is usually accompanied by T-wave alternans (Zareba et al., 1994) where the duration of the T wave can vary from one beat to the next (Jayakrishnan and Krishnakumar, 2006). This long-short alternation in duration and in some cases amplitude has been shown to arise from a period-doubling bifurcation (Nolasco and Dahlen, 1968; Guevara et al., 1984) originating at the cellular level (Pastore et al., 1999). In space, alternans can lead to complex spatiotemporal patterns along the epicardium and endocardium (Gizzi et al., 2013) and eventually to conduction block and fibrillation (Fenton et al., 2002; Choi et al., 2007).

During fast pacing, alternate patterns of action potential duration (APD) in space can be classified as concordant alternans (CA), in which all the tissue responds with a long APD on one beat and with a short APD on the following beat with the sequence repeating, or discordant alternans (DA), in which one section of tissue responds with a long APD and another with a short APD on the same beat followed by the reverse on the next beat. During DA, the regions of long and short APDs that alternate out-of-phase are separated by nodes, which are regions where the APDs have the same values for successive beats and hence do not alternate (Qu et al., 2000; Watanabe et al., 2001).

To date, two main mechanisms for the development of discordant alternans have been proposed, one driven by voltage and another by calcium (Saitoh et al., 1988, 1989). The first mechanism identified (Nolasco and Dahlen, 1968) was purely voltage-driven (Guevara et al., 1984); in space it is coupled through the dynamical interaction between the APD restitution curve and the conduction velocity (CV) restitution curve. When tissue is paced rapidly, diastolic intervals are shorter, causing slower CV near the stimulating site while CV increases downstream along wavefront propagation, causing a large spatial dispersion in the APD that can lead to DA (Qu et al., 2000; Watanabe et al., 2001).

The other mechanism, calcium-driven, is considered more complex, with DA caused by instabilities in [Ca_i_]^+2^ cycling that in turn impacts APD through [Ca_i_]^+2^–V_m_ coupling (Chudin et al., 1999; Sato et al., 2006). [Ca_i_]^+2^–V_m_ coupling depends on a dynamical balance between the influx through the L-type calcium current (I_CaL_) and extrusion through the Na-Ca exchanger current (I_NCX_) (Weiss et al., 2006). If the effect of I_NCX_ dominates, positive [Ca_i_]^+2^–V_m_ coupling will occur, where a large [Ca_i_]^+2^ causes prolonged APD by an enhanced calcium extrusion through I_NCX_. Otherwise, when a large Ca transient reduces I_CaL_ through increased calcium-dependent inactivation, APD will be shortened (Edwards and Blatter, 2014). Ca instability is another multifactorial process. The key components are the fractional Ca release from the sarcoplasmic reticulum (SR), which refers to the relation between the Ca released from the SR and the SR calcium load, and the cytosolic Ca sequestration, which refers to the efficiency of Ca removal from the cytosol through the reuptake to the SR and the extrusion through the Na-Ca exchanger (Weiss et al., 2011). In general, factors increasing fractional Ca release promote Ca alternans and factors increasing Ca sequestration reduce alternans (Edwards and Blatter, 2014). Many studies have attributed cardiac alternans to disturbances of [Ca_i_]^+2^ signaling, with APD alternans considered a secondary consequence (Eisner et al., 2006; Clusin, 2008; Laurita and Rosenbaum, 2008; Myles et al., 2008; Kanaporis and Blatter, 2015).

Alternans was observed in cardiac tissue as early as 1872 (Traube, 1872), and some of the earliest mathematical models of cardiac action potentials were able to produce such phenomena (Noble, 1962; Beeler and Reuter, 1977). However, later generations of models often failed to produce alternans (DiFrancesco and Noble, 1985; Luo and Rudy, 1991, 1994; Faber and Rudy, 2000), with more detailed species-specific models for rabbit (Puglisi and Bers, 2001; Shannon et al., 2004), dog (Winslow et al., 1999) and human (Priebe and Beuckelmann, 1998; Iyer et al., 2004; ten Tusscher et al., 2004) ventricular action potentials among them. In time, other models have been specifically designed to account for alternans (Fox et al., 2002; Mahajan et al., 2008; O'Hara et al., 2011; Sato et al., 2013).

Recently the FDA's sponsored Cardiac Safety Research Consortium (Sager et al., 2014) proposed a new initiative, the Comprehensive *in-Vitro* Pro-arrhythmia Assay (CiPA), which specifies the use of mathematical models of cardiac action potentials in the aid of pro-arrhythmic drug risk assessments. Many recently developed ionic models are complex single-cell models with a large number of variables. There exists a large variability in dynamics between them as well as failures to reproduce key physiological features when they are tested in tissue (alternans, reentrant wave dynamics, dominant frequencies, etc.). The known differences between many cell and tissue models make it imperative to validate and verify models with experiments.

Toward this end, in this study, we performed dual optical-mapping recordings with high spatial and temporal resolution for [Ca_i_]^+2^–V_m_ during discordant alternans in Langendorff-perfused rabbit hearts to better quantify the alternans mechanism as it relates to LQTS. We then used the data for validation and verification of the Sato et al. voltage-calcium rabbit cell model (Sato et al., 2013).

## Materials and methods

### Heart preparation

All experiments conform to the current Guide for Care and Use of Laboratory Animals published by the National Institutes of Health (NIH Publication No. 85–23, revised 1996), and approved by the Office of Research and Integrity Assurance at Georgia Tech. New Zealand white rabbits (2–3 kg, *n* = 8) were anesthetized with ketamine/xylazine/ace-promazine (17/9/0.9 mg/kg) and then injected with heparin (300 U/Kg). After 5 min, euthanasia was induced with 120 mg/kg pentobarbital. Hearts were then quickly removed via a left thoracotomy and perfused retrogradely via the aorta with cardioplegic solution (NaCl: 6.43 g/L, KCl: 1.19 g/L, NaHCO_3_: 0.84 g/L, MgCl·6H_2_O: 3.25 g/L, CaCl_2_: 0.13 g/L), gassed with 95% O_2_ and 5% CO_2_. Then the hearts were immersed in a chamber kept at 37.0 ± 0.3°C and perfused with Tyrode's solution (NaCl: 7.24 g/L, KCl: 0.30 g/L, NaHCO_3_: 2.02 g/L, NaH_2_PO_4_·H_2_O: 0.12 g/L, MgCl·6H_2_O: 0.14 g/L, dextrose: 0.99 g/L, CaCl_2_·2H_2_O: 0.29 g/L) gassed with 95% O_2_ and 5% CO_2_ at a pressure of about 60 mmHg maintained by a peristaltic pump. Motion was suppressed by using blebbistatin at a concentration of 3–5 mM (dissolved in DMSO at the ratio of 5 mg/mL). For imaging, the heart was stained with the voltage-sensitive dye JPW-6003 (0.4 mg dissolved in 40 μL of pure ethanol) and intracellular calcium-sensitive dye Rhod-2 (1 mg dissolved in 1 mL of DMSO).

### Optical mapping

The optical system was previously described (Fenton et al., 2009; Ji et al., 2017). Briefly, six high-power LEDs were used for excitation (LED Engin, San Jose CA). Three LEDs were used for V_m_ imaging, coupled with OD4 650/20 nm excitation filters (Edmund Optics), and the other three were used for [Ca_i_]^+2^ imaging, coupled with OD4 550/20 nm excitation filters. The operations and the intensity of the LEDs were controlled by custom-designed apparatus. Series of fluorescent images corresponding to [Ca_i_]^+2^ and V_m_ dynamics were obtained using the time-multiplexing method with a single camera (Photometric Evolve 128 EMCCD), with which the switching of the different excitation LEDs was synchronized. Fluorescence images from the anterior view (partial RV and LV) were obtained at spatial resolution of 128 × 128 pixels (full frame) at 500 fps digitized with a 16-bit dynamic range A/D.

### Stimulation protocol

External bipolar stimuli (3–5 ms, strength twice diastolic threshold) were applied from the apex or the base using a downsweep pacing protocol with the pacing cycle length (PCL) starting from 400 ms. For each PCL, 150–200 stimuli were delivered to allow the system to reach steady state. The PCL was gradually shortened with decreasing steps once alternans started to appear until the occurrence of VF or wave block at any point along the wavefront propagation, usually between 150 and 130 ms. The programming sequence was coordinated with the internal camera trigger clock using an Arduino (Uno R3) so that each pacing stimulus was delivered at a known time point when the camera started to acquire a certain frame. This method allowed us to perform image stacking (Uzelac and Fenton, 2015) once steady state was reached and to detect APD and CaD variations with temporal resolution better than the 2 ms sampling rate of the camera. We found the ability to reach faster pacing rates without inducing fibrillation was highly correlated with uniform physiological temperature across the entire heart and with smaller PCL steps, especially when the PCL was less than 160 ms. To achieve stable and uniform temperature, the heart was submerged in heated Tyrode's solution and a thermometer calibrated to the precision of 0.01°C (Thermo-Fisher) was used for temperature measurement. In case of VF, the heart was defibrillated via low-energy anti-fibrillation pacing (Fenton et al., 2009) or with cardioplegia and was allowed to recover for 30 min before performing subsequent downsweep pacing for comparison.

### Data analysis

As part of the experimental data processing, stacking (ensemble averaging) was used to obtain a high S/N ratio to avoid filtering the [Ca_i_]^+2^–V_m_ signals (Uzelac and Fenton, 2015), which degraded both spatial and temporal resolutions. For each pixel, we recorded at least 150 cycles for one pacing period, then we stacked (summed) the signals for even and odd beats, excluding the first 10 cycles at the start of each PCL to allow the heart to reach the steady state.

Alternans in voltage were quantified by measuring the action potential duration (APD) and the alternans in calcium by measuring calcium transient duration (CaD). When calculating the APD and CaD, the voltage and calcium signals were first normalized between 0 and 1 for each pixel. Then the APD was calculated using a threshold of 0.5, and the CaD was calculated using a threshold of 0.4.

### Numerical simulations

The Sato et al. (2013) model for rabbit ventricular cells in space was used under conditions similar to those of the rabbit experiments. Briefly, each cardiac cell of a one-dimensional cable of tissue is modeled by 75 sarcomeres connected through the diffusion of cytosolic calcium (Cai) and network sarcoplasmic reticulum (NSR) calcium. The diffusion strengths are 8 × 10^−9^ cm^2^/ms and 4 × 10^−10^ cm^2^/ms, respectively. Voltage in the 75 sarcomeres within one cell is considered to be the same due to the fast diffusion inside a cell. Each sarcomere consists of four compartments: cytosol, submembrane, NSR and junctional SR (JSR). The calcium fluctuation is model by the Langevin equation with a noise term depending on the number of SERCA pumps.

One-dimensional tissue is modeled by the cable equation:

(1) $$\begin{array}{r} \frac{\partial V}{\partial t}=-\frac{I_{ion}}{C_{m}}+D_{V}\frac{\partial^{2}V}{\partial x^{2}}, \end{array}$$

where *C*_*m*_ is the membrane capacitance (1 μF/cm^2^), *D*_*v*_ is the voltage diffusion coefficient (10^−3^ cm^2^/ms), and *I*_*ion*_ is the total transmembrane current, which is the sum of all the ionic currents:

(2) $$\begin{array}{r} I_{ion}^{i}=I_{Na}^{i}+I_{K}^{i}+\sum_{j=1}^{M}\left(I_{Ca}^{i,j}+I_{NaCa}^{i,j}\right) \end{array}$$

Here $I_{ion}^{i}$ is the ionic current (Sato et al., 2013). Index *i* is the cell index in the 1D cable and index *j* is the index for the *j*th sarcomere in the *i*th cell. M is the total number of sarcomeres in one cell (i.e., 75 in our simulations). The cable equation is integrated using an operator splitting approach with Δx = 0.015 cm and Δt = 0.1 ms. The diffusion of calcium between cells is considered negligible.

Three different pacing protocols were used for the 1D simulations, with each using a stimulus current applied to the first five cells for a duration of 2 ms. In the first pacing protocol, we started by pacing with PCL = 600 ms until steady state was reached, then decreased the PCL to 300 ms and paced until steady state was reached. In the second pacing protocol, we initially assigned to the whole cable the steady state variables for the leftmost cell for PCL = 600 ms, then we gradually decreased the PCL to 300, 295, 290, 285, 280, 270, and 260 ms. For PCLs longer than 300 ms, we used the third pacing protocol, in which we used the steady state of PCL = 300 ms from the second protocol as the initial condition. When calculating APD, −80 mV (about 80% repolarization) was used as the threshold. When calculating CaD, the threshold was set to be between 10 and 20% of the repolarization, adjusted among different pacing cycle lengths to make sure both even and odd beats can be captured.

## Results

### [Ca]_i_ alternans develops at longer PCLs than APD alternans

**Figure 1:**
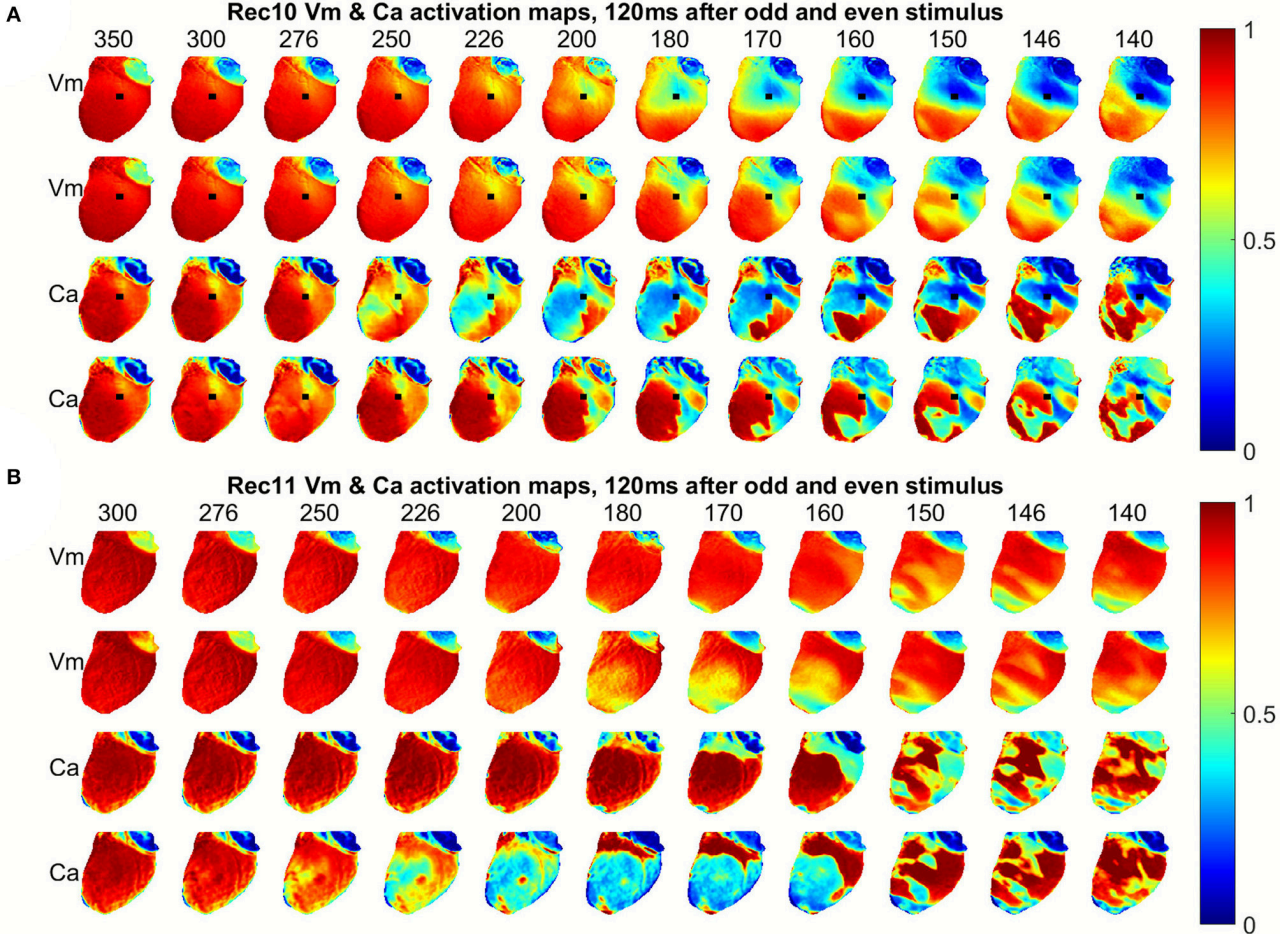
Snapshots of normalized voltage (upper two rows) and intracellular Ca (lower two rows) 120 ms after stimulus application on consecutive beats for decreasing pacing cycle lengths (PCL) from 350 to 140 ms (left to right). Stimulus is applied at **(A)** the base and **(B)** the apex.

**Figure 2:**
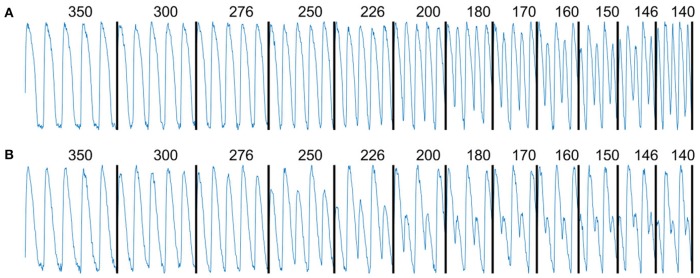
Time traces of normalized voltage **(A)** and [Ca]_i_ **(B)** signals from a single pixel marked in Figure 1A for the same PCLs.

The spatiotemporal dynamics of voltage and calcium in cardiac tissue depends on the pacing period. Figure 1A shows snapshots of voltage (upper two rows) and calcium (lower two rows) in a rabbit ventricle for a series of PCLs from 350 to 140 ms when stimulation was applied at the base of the heart (black arrow). Each column shows consecutive even and odd images during steady state 120 ms after stimulus application; all frame over two successive beats at steady state are shown in Supplementary Movie 1. Figure 1B shows the same situation but when the stimulus is applied to the apex, and Supplementary Movie 2 shows all frames over two successive beats at steady state for this case. In all rabbit experiments, Ca alternans clearly developed at longer PCLs than voltage alternans. As the tissue was paced more rapidly, inhomogeneity emerged in both voltage and calcium patterns with calcium displaying more spatial heterogeneity compared to voltage. Figures 2A,B shows the voltage and calcium signals, respectively, over time for one pixel indicated by a marker in Figure 1 for all PCLs. As the PCL decreases, alternans was detected first in calcium amplitude (250 ± 10 ms), then in calcium duration (220 ± 15 ms), and finally in APD (200 ± 15 ms). The shortest PCLs can barely generate an excitation in calcium for the short beats (last two panels in Figure 2B), and alternans is more pronounced in both duration and amplitude for the calcium signal.

### Discordant alternans nodes are more pronounced in [Ca]_i_ than in APD

**Figure 3:**
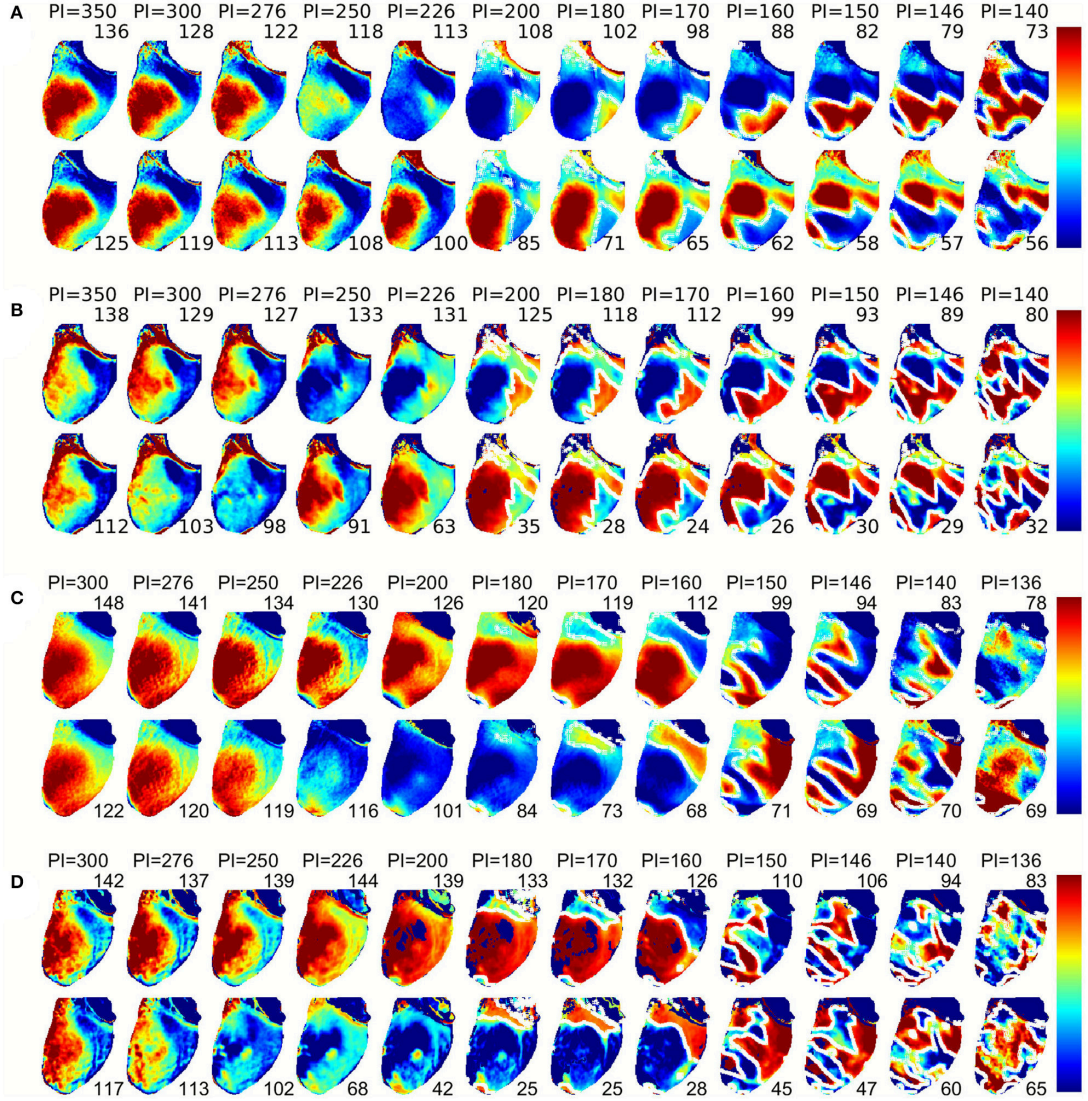
**(A)** Spatial distribution in APD for even (top) and odd (bottom) beats at various PCLs with stimulation applied to the base. Numbers next to the maps indicate maximum and minimum values used in the color map in ms. Notice there is no alternans for PCL > 275 ms, concordant alternans for PCL between 250 and 225 ms, and discordant alternans for PCLs below 225 ms. Nodal lines are shown in white. **(B)** [Ca]i spatial distribution for even (top) and odd (bottom) beats as in **(A)** but transitions between discordant alternans phases are sharper and nodes are more pronounced. **(C,D)** Spatial distributions as in **(A,B)** but for pacing from the apex.

Figure 3A shows the spatial dispersion of APD for successive beats at steady state. Several important features are worth noticing. At long PCLs (e.g., >250 ms), there is no difference between even and odd beats (no alternans), but there is an intrinsic smooth spatial dispersion of APDs in the range of 15 ms for each beat. As concordant alternans and then discordant alternans develop, the gradient of APD increases to around 30 ms as the PCL decreases. The distance between the locations of the longest and shortest APD decreases during DA, similar to what has been observed in canine hearts (Gizzi et al., 2013). During DA, the regions of long-short and short-long APD are separated by a collection of nodes (nodal lines) where the APD remains constant from beat to beat (shown as white lines in Figure 3), with more nodes forming as the PCL decreases. Figure 3B shows the CaD dispersion in tissue, similar to Figure 3A. However, during DA, the nodal lines are thicker and more pronounced compared to voltage nodal lines. Figures 3C,D shows plots similar to Figures 3A,B for the same preparation but with the pacing site located at the apex instead of the base. The progression from no alternans to CA and then to DA occurs at the same PCLs, but the spatial patterns are different. This difference of patterns depending on pacing site was observed in all 8 rabbit experiments as well as in previous studies using canine hearts (Gizzi et al., 2013).

**Figure 4:**
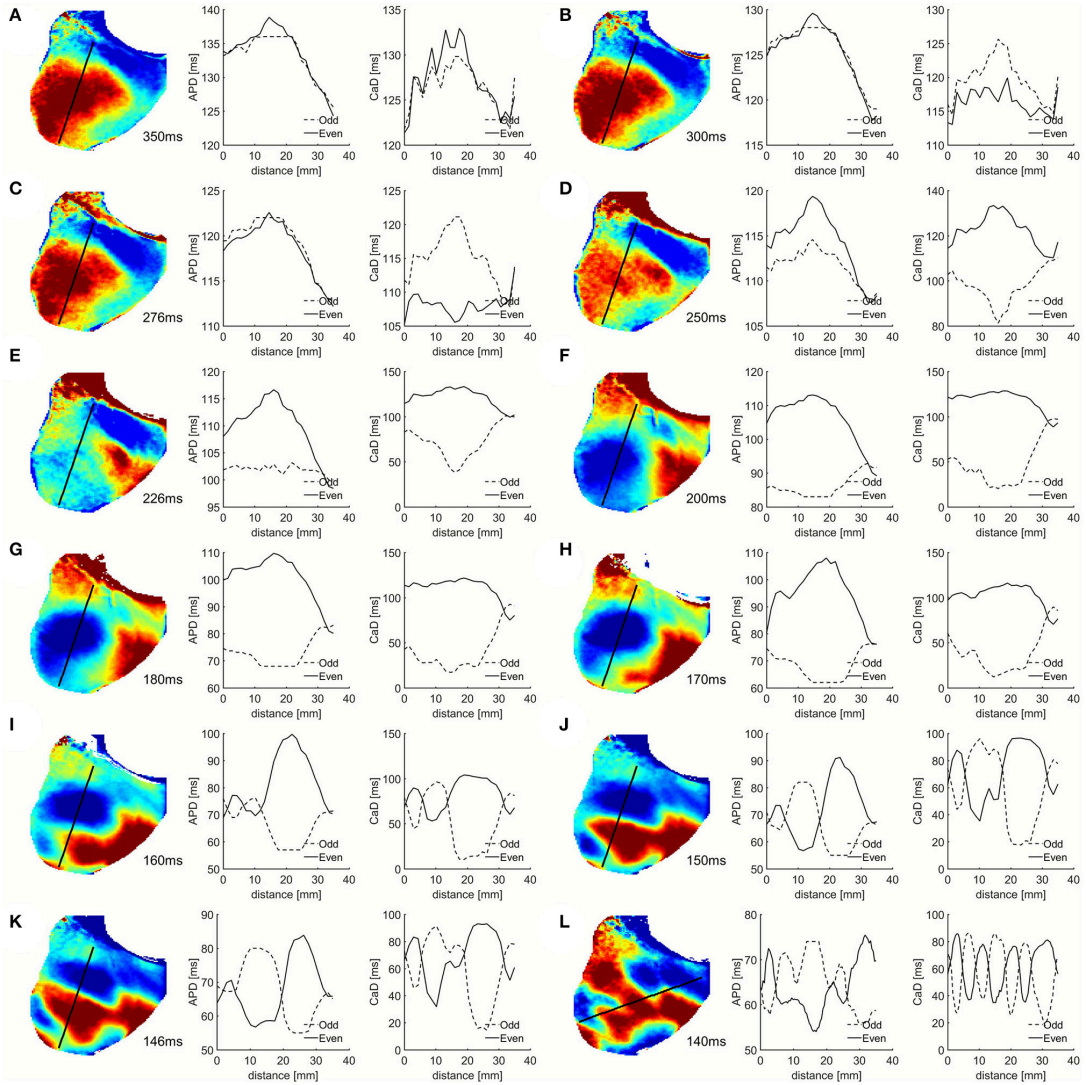
APD contour and APD and [Ca]_i_ alternans duration in ms for even and odd beats measured along a one-dimensional line shown in black for different PCLs as in Figure 3.

Previous numerical studies of alternans (Qu et al., 2000; Watanabe et al., 2001; Fenton et al., 2002; Skardal et al., 2014) have used one-dimensional cables to quantify the spatial profiles of APDs, including the number of nodes present. In the same way, Figure 4 displays the values of APD and CaD along a line across the heart's surface for two successive beats during alternans. As in Figure 3, it can be seen that there is no alternans for PCL >250 ms, then concordant alternans in voltage and calcium appears for PCLs between 200 and 180 ms. At 170 ms, there is CA in voltage but DA for calcium, and for PCL < 170 ms DA is present for both voltage and calcium. We calculated in Table 1 the steepness (slope) of the APD and CaD nodal lines for PCL between 160 and 146 ms corresponding to Figures 4I–L when the DA are most pronounced. Data was presented as mean ± s.d., averaged among the slopes at each node for even and odd beats for each pacing cycle length. It clearly shows that CaD nodal lines are about one order of magnitude steeper than APD nodal lines, indicating the diffusive connection among cells differs in voltage and calcium signals (Shiferaw and Karma, 2006; Gaeta et al., 2009), as the lack of diffusion in calcium leads to calcium profiles that are sharper in space compared to voltage.

**Table 1:** Steepness of APD and CaD nodal lines.

	**PCL = 160 ms**	**PCL = 150 ms**	**PCL = 146 ms**	**PCL = 140 ms**
Steepness of APD nodal lines (ms/mm)	2.20 ± 0.87	3.11 ± 1.54	2.75 ± 1.51	1.12 ± 0.38
Steepness of CaD nodal lines (ms/mm)	12.1 ± 4.8	12.5 ± 3.7	9.94 ± 2.91	20.1 ± 5.6

### Experimental alternans features smoother spatial profiles and slower alternans amplitude growth than simulated alternans

**Figure 5:**
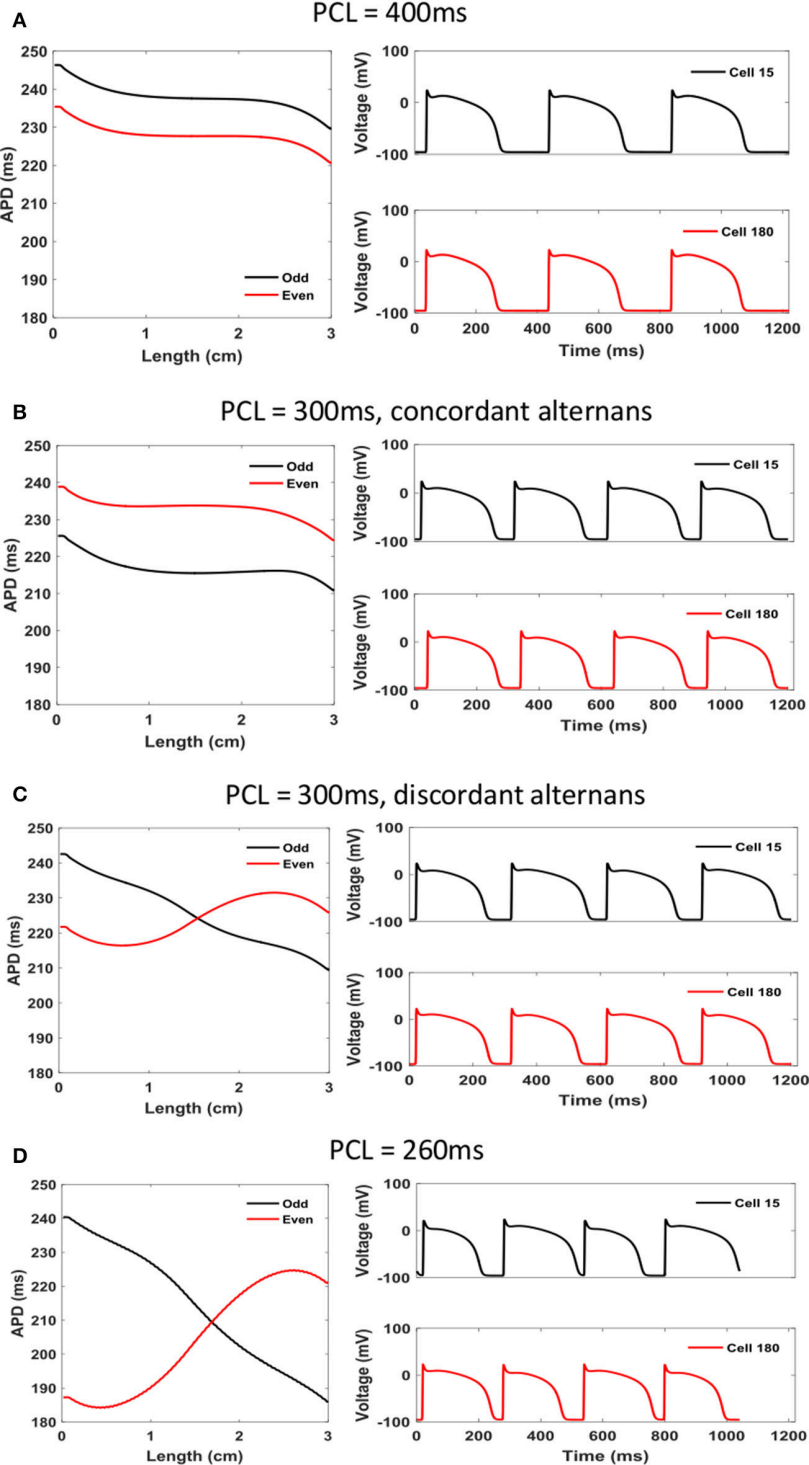
Voltage alternans in a 1D cable (*L* = 3 cm, 200 cells) of the Sato et al. model for decreasing PCLs: **(A)** PCL = 400 ms, **(B)** PCL = 300 ms (concordant alternans), **(C)** PCL = 300 ms (discordant alternans), and **(D)** PCL = 260 ms (discordant alternans). Left: spatial profile of APD for odd (black) and even (red) beats. The amplitude of discordant alternans increases as PCL decreases. Right: voltage over time at two cells, one near the left end and the other near the right end of the cable.

**Figure 6:**
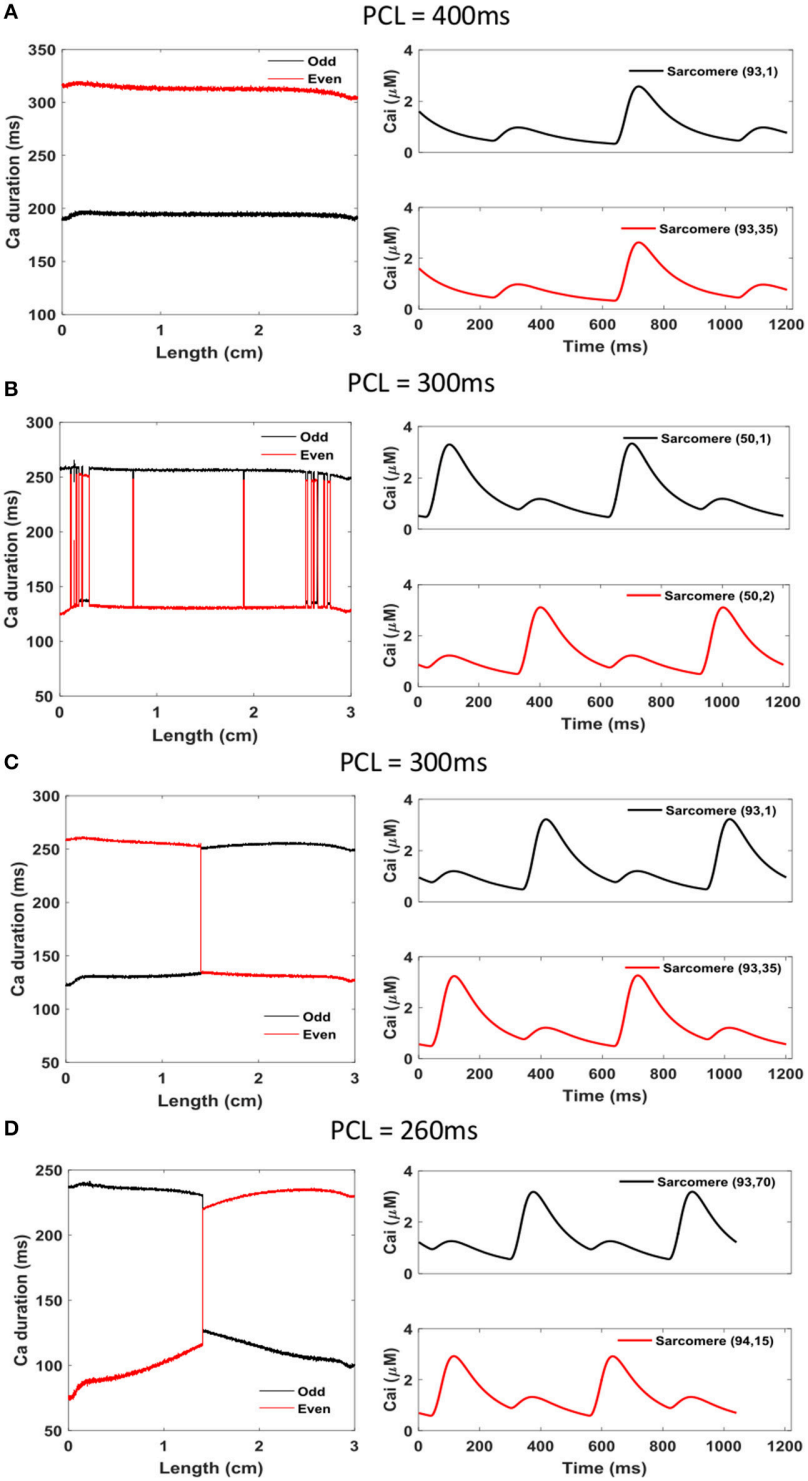
Calcium alternans in a 1D cable (*L* = 3 cm, 200 cells, each cell consists of 75 sarcomeres). **(A)** PCL = 400 ms, **(B)** PCL = 300 ms, **(C)** PCL = 300 ms, and **(D)** PCL = 260 ms. Left: spatial profile of Ca duration for odd (black) and even (red) beats. Right: Ca over time at two sarcomeres that are around the CaD nodal point where they are completely out of phase when discordant alternans is present.

Simulation results using the Sato et al. model confirm that alternans appear as the PCL is decreased. However, the PCLs at which they appear are much longer compared to experiments. Figures 5, 6 show voltage and calcium alternans in a 1D cable 3 cm in length. Figure 5 illustrates APD as a function of length in the left column for even and odd beats at PCLs of 400, 300, and 260 ms. The right column shows the voltage signal for the corresponding PCL for two cells, one near the left end and one near the right end of the cable, so that if it undergoes discordant alternans, the voltage signal of the two cells should be out of phase. Figure 6 shows similar plots but for calcium. The left column indicates CaD over the cable for even and odd beats for the same PCLs as in Figure 5. The right column of Figure 6 displays the calcium signal from two sarcomeres for corresponding PCLs, with the two sarcomeres chosen so that if there is discordant alternans in calcium, the two sarcomeres should be out of phase. Results for other PCLs (600, 500, 450, 350, 290, 280, and 270 ms) are presented in Supplementary Figure 1 for APD and Supplementary Figure 2 for CaD.

For large PCLs (>400 ms), there is no alternans in either voltage or calcium (Supplementary Figures 1A–C, 2A–C). When the PCL drops to 400 ms, concordant alternans appears (Figures 5A, 6A). For PCLs 300 ms or below, discordant alternans occurs (Figures 5C,D, 6C,D and Supplementary Figures 1E–G, 2E–G). In all these simulations, voltage and calcium alternans are “synchronized,” such that if there is no voltage alternans, there is no calcium alternans, and if there is discordant voltage alternans, there is discordant calcium alternans, with the exception of Figures 5B, 6B. In Figures 5B, 6B, where we used the second pacing protocol and the PCL is the same as Figure 5C, we obtained concordant voltage alternans, whereas the calcium alternans is discordant with multiple nodes. By using different initial conditions, it is possible to obtain completely different dynamics for the same PCL. The Sato et al. model is very sensitive to initial conditions due to the random fluctuation term in the SERCA pump, and in this respect, it is similar to experiments, where it has been shown that small changes in initial conditions can result in very different alternans patterns (Gizzi et al., 2013). In all the simulations presented in this paper, the random noise was generated using the same seed. We did not observe significant changes when we repeat the simulations with different seeds (data not shown).

One major difference between the experiments and the simulations is how sharp the calcium transition is between different alternans phases. In the experiments, both APD and CaD have relatively similar smooth transitions between the maximum and minimum values (Figures 3, 4), with calcium showing only a slightly faster transition, whereas in simulations, CaD has a significantly sharper transition than APD (Figures 5, 6). In addition, in experiments, as the PCL is decreased, CaD transitions from no alternans to concordant alternans and then to discordant alternans with the alternans amplitude increasing smoothly. In simulations, on the other hand, CaD changes drastically from no alternans to concordant alternans with an amplitude of 150 ms when the PCL decreases from 450 to 400 ms.

**Figure 7:**
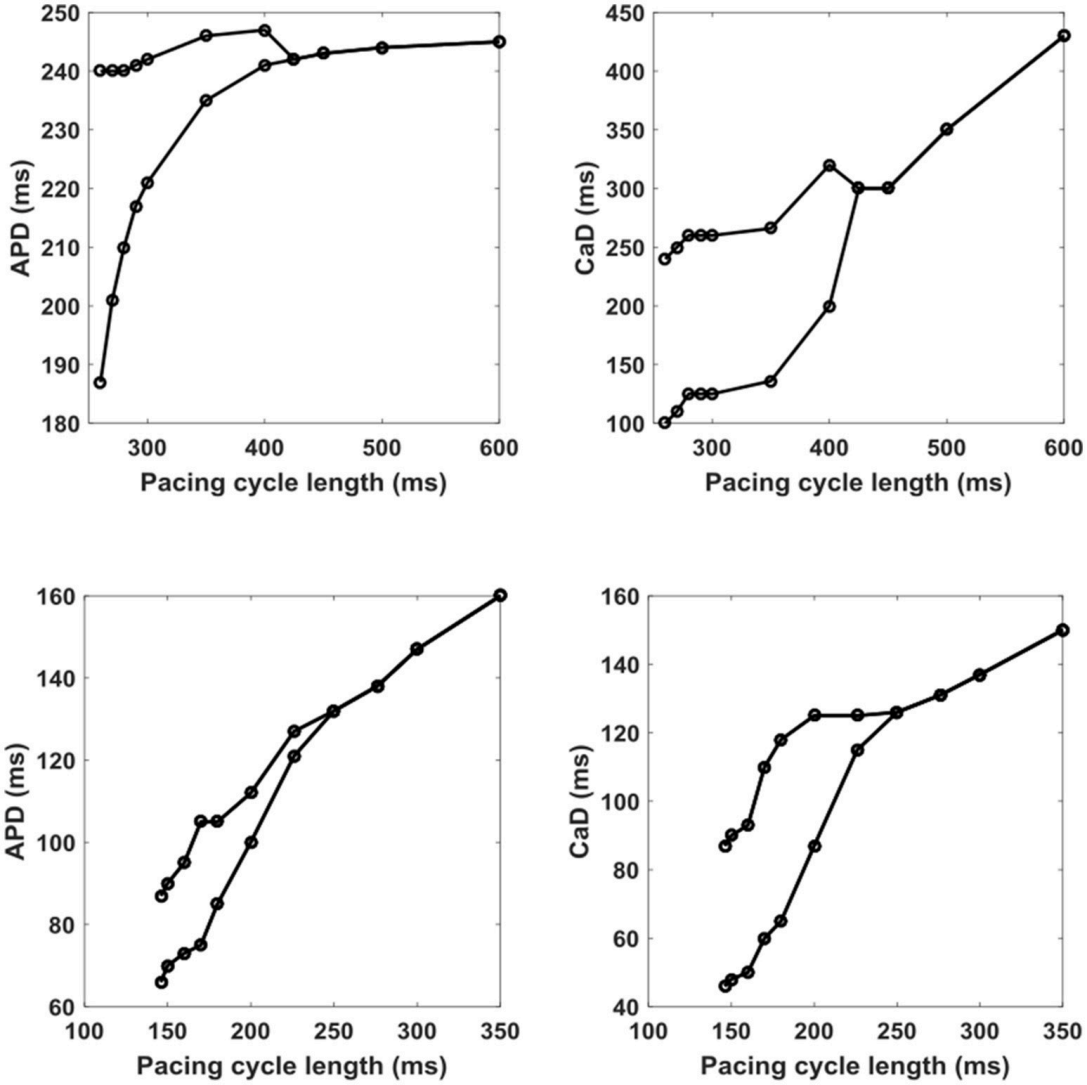
Bifurcation plots of APD **(Left)** and CaD **(Right)** as a function of PCL for simulations (top) and experiments (bottom).

Figure 7 shows a bifurcation diagram for APD (left) and CaD (right) as a function of PCL calculated at one point from the numerical simulation of the 1D cable (top) and experimental data (bottom) where the alternans has the largest amplitude. As the PCL is decreased from 600 to 260 ms in the numerical simulations, the bifurcation appears simultaneously for both voltage and intracellular calcium just above a CL of 400 ms (PCL_c_). The bifurcation amplitude for voltage grows at a rate of the square root away from the bifurcation point (ΔAPD ~ (PCL − PCL_c_)^1/2^) (Cherry and Fenton, 2008). The bifurcation amplitude in calcium experiences a sharp discontinuous jump (see Supplementary Figure 3 for the curve fitting). In contrast, the experimental bifurcations occur at much lower PCLs close to 250 ms, and the amplitude of alternans just beyond the bifurcation can be fit better into a linear function with a smoother growth than in the simulations.

## Discussion

Patients with LQTS have a higher risk of cardiac arrhythmias due to augmentation of the T-wave and increased spatial dispersion. For many years now, detection of LQTS and T-wave alternans in the ECG has been used as a quantitative tool for predicting dangerous spatial variations in dispersion, which are dynamically induced at the cellular level and can induce arrhythmias. Since 2005, the FDA has required that new drugs be tested for QT prolongation and development of T-wave alternans and recently an FDA-sponsored consortium proposed an initiative to use mathematical action potential models in the aid of drug risk assessment. If models are indeed to be used to investigate pro-arrhythmic and anti-arrhythmic effects of drugs, they first need to be validated against experimental data in normal conditions. The main goal of this study is to create an in tissue experimental data set of simultaneous voltage and calcium optical mapping recordings from Langendorff-perfused rabbit hearts at high temporal and spatial resolution and in particular during alternans for use in validating the dynamics from numerical simulations from the most recent model of rabbit ventricular action potentials (Sato et al., 2013).

We found that alternans in voltage and calcium develops similarly in both experiments and simulations as the pacing cycle length is decreased; however, the model developed alternans much sooner at longer periods close to 420 ms compared to 240 ± 10 ms in experiments. Likewise, the minimum period of stimulation before conduction block developed earlier at 260 ms in the model vs. 140 ± 5 ms in the experiments. Also, it is important to notice that in experiments, as in the models, the magnitude in variations of the APD were much smaller than the variations in CaD during alternans. However, the magnitudes in alternans duration were about twice as large in the model as in the experiments, with maximum values of 55 ms for APD and 150 ms for CaD in the model vs. 25 ± 4 and 65 ± 5 ms, respectively, for the experiments.

More crucial differences arise from the larger differences in how alternans develops. In experiments, alternans in voltage develops gradually, appearing to be more consistent with a border-collision bifurcation (Cherry and Fenton, 2007, 2008; Zhao et al., 2008), where small changes in duration grow slowly and mostly linearly, whereas in the model alternans grows much faster, as with a pitchfork bifurcation (Cherry and Fenton, 2008). Furthermore, the model does not yield action potential amplitude (APA) alternans as observed in the experiments when the tissue is paced at very short pacing cycle lengths (see Supplementary Figure 4 for the APA bifurcation map from experiment), an important additional pro-arrhythmic mechanism recently described by Myles et al. (2011); Chen et al. (2017). On the other hand, while intracellular calcium does display amplitude alternans in both experiments and simulations, in experiments amplitude alternans develops slowly, while in the model a large difference in amplitude appears as soon as alternans develops and persists with a similar amplitude for all periods where alternans is present, as shown in Figure 6.

In regard to spatial distributions, in our experiments, we do not detect very sharp calcium duration transitions around nodal lines for most pacing cycle lengths; instead, we observe a smooth phase transition in both voltage and calcium, albeit with calcium appearing sharper, with more defined nodal lines than those in APD (Figure 3 and Table 1). This is contrary to simulations where CaD alternans transition can happen within a cell. If the coupling between voltage and calcium is strong, calcium and voltage should have similar dynamics, i.e., the CaD alternans nodal line should follow that of the APD and vice versa. Therefore, we think the model, as it was published, lack necessary coupling between voltage and calcium. The resolution of our optical mapping is on the order of 200–250 microns; however, due to scattering (Bishop et al., 2007), the actual area may be smoothed to about a millimeter, which is still high enough to identify any possible sharp differences in heterogeneities between voltage and calcium. It is then possible that during pacing in tissue, calcium nodal lines still follow the smoother dynamics of voltage compared to when it is performed in single cell experiments (Gaeta et al., 2009).

### Shortcomings of the model

While the PCL at which alternans appears is much higher in the model than in the experiments by approximately 180 ms, this difference could be fixed with a simple re-scaling of some of the time constants of the model. Similarly, the difference between the minimum PCL for propagation (around 260 ms for the model compared to 140 ms for experiments) could also be fixed by modifications to the recovery and inactivation time constants of the sodium gating variables. Previous publication also showed that CaD alternans patterns can be smoothed by increasing voltage instability (and/or reducing Ca instability). However, there exist several other key physiological aspects that would require a more in-depth analysis and validation of the model's equations.

- Type of bifurcation. The calcium dynamics should represent the fast nonlinear transition in the dynamical content of calcium in the sarcoplasmic reticulum (Díaz et al., 2004) that can result in a border-collision bifurcation with slow linear growth.
- Development of alternans in calcium duration. In the model, as soon as alternans develops in calcium, its amplitude is almost at its maximum (Figure 6), whereas in the experiments, there is a very smooth transition in Ca alternans with decreasing cycle length (Figure 2B).
- Development of alternans in amplitude. In the model, when alternans in the AP duration develops, at no point does alternans in AP amplitude appear (Figure 5). This is in contrast to experiments, where at very short cycle lengths alternans in amplitude is readily observed (Figure 2A). Recent experiments and theory predict that the presence of amplitude alternans is key in the development of reentrant arrhythmias (Myles et al., 2011; Chen et al., 2017).
- CV and nodal lines. In both simulations and experiments, concordant and discordant alternans appears in tissues of similar size of about 3 cm. The conduction velocities are very different between simulations having a maximum velocity at about 47 cm/s, vs. experiments at around 100 cm/s. Numerically, this means that if the model's diffusion coefficient was modified to fit the experimental CV values, it will result in tissues of more than twice the size needed experimentally to support discordant alternans.

### Limitations

The mechanical and electrical behaviors of the heart are strongly coupled through calcium signaling. Very few mathematical models incorporate both aspects (Ji et al., 2015) even though there exists a strong bi-directional coupling between them. We did not study the effect of alternans on contraction or vice versa in either experiments or simulations and we did not study the effect of temperature on calcium and voltage alternans. It has been shown that mammalian hearts can largely increase the magnitude of alternans when temperature is lowered (Pastore et al., 1999; Fenton et al., 2013; Filippi et al., 2014).

## Disclosure

The mention of commercial products, their sources, or their use in connection with material reported herein is not to be construed as either an actual or implied endorsement of such products by the Department of Health and Human Services.

## Author contributions

IU: experiment, data analysis and writing. YJ and EC: simulation and writing. DH, JS, SL, amd RG: experiment. FF: experiment, simulation and writing.

### Conflict of interest statement

The authors declare that the research was conducted in the absence of any commercial or financial relationships that could be construed as a potential conflict of interest. The reviewer DS and handling Editor declared their shared affiliation, and the handling Editor states that the process met the standards of a fair and objective review.
