# Supplementary material for: Simultaneous Quantification of Spatially Discordant Alternans in Voltage and Intracellular Calcium in Langendorff-Perfused Rabbit Hearts and Inconsistencies with Models of Cardiac Action Potentials and Ca Transients
Source: Front Physiol. 2017 Oct 20;8:819. doi: 10.3389/fphys.2017.00819 (PMC5655020; doi:10.3389/fphys.2017.00819)
Supplement: Supplementary file 3 [file DataSheet1.DOCX]

Supplementary Material

Simultaneous Quantification of Spatially Discordant Alternans in Voltage and Intracellular Calcium in Langendorff-perfused Rabbit Hearts and Inconsistencies with models of cardiac action potentials and Ca transients

Ilija Uzelac, Yanyan Claire Ji, Daniel Hornung, Johannes Schrӧder-Scheteling, Stefan Luther, Richard A. Gray, Elizabeth M. Cherry and Flavio H. Fenton^*^

*** Correspondence:**

**Flavio H. Fenton**

[Flavio.fenton@physics.gatech.edu](mailto:Flavio.fenton@physics.gatech.edu)

## Supplementary Figures

**Supplementary Figure 1.** APD for different pacing cycle lengths.

1. PCL = 600ms
2. PCL = 500ms
3. PCL = 450ms
4. PCL = 350m
5. PCL = 290ms
6. PCL = 280ms
7. PCL = 270ms

**Supplementary Figure 2**. CaD for different pacing cycle lengths

1. PCL = 600ms
2. PCL = 500ms
3. PCL= 450ms
4. PCL= 350ms
5. PCL= 290ms

(F) PCL = 280ms

(G) PCL = 270ms

**Supplementary Figure 3. Bifurcation amplitude of APD (left) and CaD (right) of experiments (top row) and simulations (bottom row).** We can fit the bifurcation amplitude beyond the bifurcation point in the experiment into a linear function (ΔAPD/ ΔCaD ~ (PCL – PCL_c_)) with r square of 0.9306 and 0.9739 for voltage and calcium respectively. The voltage bifurcation amplitude in simulation can be fit into a function ΔAPD ~ (PCL – PCL_c_)^1/2^ with r square of 0.9802.

**Supplementary Figure 4. Action potential amplitude bifurcation from data in Figure 2A**

**
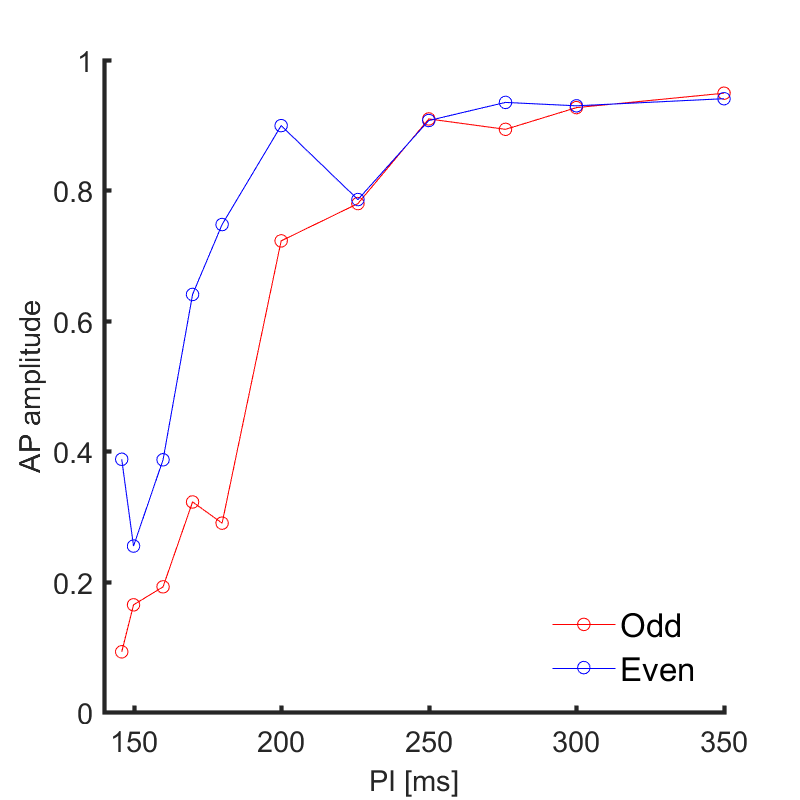
**

## Supplementary Movies

**Supplementary Movie 1.** Voltage and calcium in two consecutive beats for pacing cycle lengths from 350 to 140 ms when stimulation was applied at the base of the heart.

**Supplementary Movie 2.** Voltage and calcium in two consecutive beats for pacing cycle lengths from 350 to 140 ms when stimulation was applied at the apex of the heart.
